# Effects of yoga on oxidative stress, motor function, and non-motor symptoms in Parkinson’s disease: a pilot randomized controlled trial

**DOI:** 10.1186/s40814-018-0355-8

**Published:** 2018-10-23

**Authors:** Corjena Cheung, Rozina Bhimani, Jean F. Wyman, Jürgen Konczak, Lei Zhang, Usha Mishra, Marcia Terluk, Reena V. Kartha, Paul Tuite

**Affiliations:** 1 School of Nursing, University of Minnesota, Minneapolis, MN 55455 USA; 2 School of Kinesiology, University of Minnesota, Minneapolis, MN 55455 USA; 3 Clinical and Translational Science Institute, University of Minnesota, Minneapolis, MN 55455 USA; 4 Center for Orphan Drug Research, Department of Experimental & Clinical Pharmacology, University of Minnesota, Minneapolis, MN 55455 USA; 5 Department of Neurology, University of Minnesota, Minneapolis, MN 55455 USA

**Keywords:** Parkinson’s disease, Yoga, Oxidative stress, Motor function, Non-motor symptoms

## Abstract

**Objective:**

To examine the feasibility, acceptability, and preliminary effects of Hatha yoga on oxidative stress, motor function, and non-motor symptoms among individuals with Parkinson’s disease (PD).

**Methods:**

The study has a pilot randomized controlled trial design with two arms: an immediate treatment group and a wait-list control group. The yoga-for-PD program was implemented via twice weekly 60-min group-based classes for 12 weeks. Participants were assessed at baseline, 12 weeks, and 6 months post-intervention. Outcome measures included oxidative stress, motor function, physical activity, cognitive function, sleep quality, and quality of life. Data on program acceptability and yoga adherence were collected during the intervention and at 6 months post-intervention.

**Results:**

Participants (*n* = 20) had a mean age of 63 years (SD 8, range 49–75) and disease duration 4.8 years (SD 2.9, range 1–13). All participants had mild-moderate disease severity; 18 (90%) were on dopaminergic medications. Seventeen participants (85%) attended at least 75% of the classes and 4 (20%) attended all classes. Most participants (*n* = 17) reported they “definitely enjoyed” the intervention program. No adverse events were reported. At 12 weeks, there were no major differences in blood oxidative stress markers between the two groups. Motor function based on the Unified Parkinson’s Disease Rating Scale was better in the treatment group, but their scores on sleep and outlook in Parkinson’s Disease Quality of Life (PDQUALIF) Scale and the physical activity levels based on the Longitudinal Aging Study Amsterdam Physical Activity Questionnaire were worse than those of the control group. In within-group comparisons, motor function, cognitive function, and catalase improved but three PDQUALIF domains (social and role function, sleep, and outlook) and physical activity level worsened by the end of the yoga intervention program compared to baseline. The response rate for the 6-month follow-up survey was 74% (*n* = 14) with six participants (43%) who signed up for a yoga class and four (29%) who practiced it independently. Health problems were the main barrier to yoga practice.

**Conclusion:**

Yoga is feasible and acceptable and may serve as a complementary method for improving motor function in PD. Further research using a larger sample size is needed to determine its impact on oxidative stress and non-motor symptoms.

**Trial registration:**

ClinicalTrials.gov Registration Number: NCT02509610031.

## Introduction

Parkinson’s disease (PD) is a common age-related neurodegenerative disorder that affects over 10 million people worldwide [1]. In the USA alone, approximately 60,000 new cases of PD are diagnosed each year with the annual treatment cost estimated to be $25 billion [2]. While loss of dopamine is crucial to the manifestation of the cardinal features of PD (bradykinesia, postural instability, rigidity, and resting tremor), symptomatic dopaminergic therapies address only some of its motor and non-motor impairments [3] and do not prevent or treat an assortment of gait difficulties [4].

Although the pathology of PD is complex, oxidative stress is thought to play a key role in the progressive loss of dopaminergic neurons in the substantia nigra of the brain [5]. Oxidative stress is defined as a disturbance in the balance between the production of reactive oxygen species (free radicals) and antioxidant defenses. It occurs as a result of an alteration in the equilibrium of the production of reactive oxygen species and antioxidative processes within the body [6]. Dopaminergic neurons are particularly vulnerable to oxidative stress because dopamine metabolism and transport can impact reactive oxygen species production [7]. Postmortem analysis revealed excessive production of reactive oxygen and nitrogen species and decreased levels of the antioxidant glutathione (GSH; biomarker of oxidative stress) in the substantia nigra, providing evidence for oxidative stress in PD [5].

Exercise is an integral part of the management of PD because physical activity has been shown to reduce oxidative stress [8], delay the deterioration of motor functions [9], and improve mood impairments [10]. However, traditional aerobic or resistance-based exercises require safety monitoring, and some are equipment-dependent. Hatha yoga is commonly used by the general public as a form of exercise in North America. Because of its gentle approach, yoga shows promise as an intervention that can be adapted to persons with PD who may not be able to participate in strenuous or intensive exercise. The theory underlying yoga practice is that the union of mind and spirit in exercise brings balance to the body and promotes healing [11]. The practice of Hatha yoga, which incorporates poses, breathing techniques, and meditation, has documented health benefits (e.g., flexibility, strength, and relaxation) and can maintain or improve the antioxidant level of the body in young healthy individuals [12] and individuals with type II diabetes, hypertension, and end-stage renal disease [13–15].

Despite the well-known benefits of yoga in selected chronic disease populations, evidence on the effect of yoga in persons with PD is limited. Four randomized controlled trials (RCT) of yoga in PD patients were located [16–19]. Although all showed positive therapeutic benefit of yoga in managing motor function and non-motor functions, these studies had methodological limitations including the lack of blinding [19], randomization issues [17], small sample sizes [16–18], and none examined oxidative stress as an outcome or long-term yoga adherence. The purpose of this pilot RCT study was to examine the feasibility, acceptability including yoga adherence after the intervention, and preliminary effects of Hatha yoga on oxidative stress, motor function, and non-motor symptoms among individuals with PD. We hypothesize that Hatha yoga will provide symptomatic and disease-modifying effects via decreasing oxidative stress.

## Methods

### Design

This pilot study used a RCT design with two arms: an immediate treatment group who received a 12-week Hatha yoga program and a wait-list control group. The allocation ratio was 50/50. Outcomes were assessed at multiple time points: baseline prior to initiation of the yoga intervention program, 12 weeks upon the completion of the intervention program, and 6 months post intervention. Because participants in the wait-list control group received the same intervention program after the treatment group completed their program, the wait-list control group had two baseline measurements: at the beginning of study (first) and at 12 weeks prior to their intervention program (second). A 24-week data was collected from the wait-list group upon the completion of the intervention program. There was a total of four data collection points for the wait-list group. The research protocol was approved by the University of Minnesota Institutional Review Board.

### Participants and randomization

**Fig. 1:**
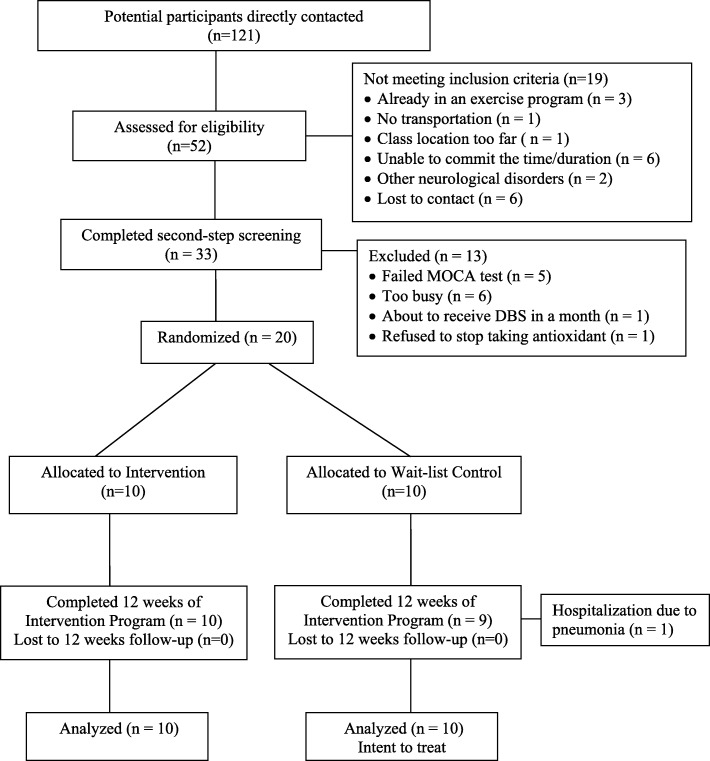
CONSORT flow diagram of study participants

The CONSORT flow diagram (Fig. 1) illustrates the recruitment and retention process for this study. The sample size was determined by the feasibility of having no more than 10 participants in class with one yoga teacher and a research assistant (RA). Participants were recruited from clinics via flyers, through local and national PD networks such as PD support groups and PD community events, and through the study website or were referred by an investigator from his neurological practice. Inclusion criteria were as follows: individuals diagnosed with mild to moderate idiopathic PD (Hoehn and Yahr stages I–III) [20], age 45–75 years, on stable dopaminergic therapy for 4 weeks prior to enrollment if taking medication, and able to ambulate 6 m with/without assistive device. Individuals were excluded if they had atypical parkinsonism or other significant brain conditions such as a stroke, had any medical condition that prohibited safe exercise as assessed by the Exercise Assessment and Screening for You Questionnaire [21], had significant cognitive impairment as indicated by scoring less than 26 in the Montreal Cognitive Assessment (MoCA) [22], had a decline in immune function such as pneumonia or systemic infection, had spinal fusion or other orthopedic surgery in the past 6 months, had a significant psychiatric disease, needed greater than minimal assistance for gait and transfers, were already practicing yoga regularly, or were unable to commit to attend scheduled yoga sessions. The number of potential participants assessed for eligibility was documented to give an indication of the appeal of the yoga program. Informed written consent was obtained just prior to the initial assessment.

Each participant was assigned a study identification (ID) number from 1 to 20 based on the order of enrolment. A computer-generated random assignment list prepared by a statistician was used to randomize participants. Group allocations were contained in a set of sealed envelopes, each bearing on the outside only the study ID number. The envelopes were distributed by a RA at the end of baseline data collection. Participants assigned to the treatment group participated immediately in a 12-week Hatha yoga group intervention program. Participants in the wait-list group served as control during the first 12 weeks and received the same intervention afterward. The RA who collected the data, the scientists who performed the laboratory analyses, and the statistician who performed the data analysis were blinded to group assignment.

### Interventions and setting

The yoga for PD intervention program was initially designed by the lead yoga instructor based on a focused literature review on relevant yoga programs. The draft program was reviewed by an expert panel composed of six yoga experts who specialized in teaching individuals with musculoskeletal and neurological disorders. The expert panel met for a 2-h meeting to discuss the feasibility and intended effects of the program. The final program was approved by all the experts and implemented in the study [23]. The yoga intervention sessions were held twice weekly for 60 min each session at a local yoga studio which was conveniently located at street level with ample parking space. For safety reasons, home practice was not prescribed in this pilot project.

### Outcome measures

The primary outcome of the study was the alterations in oxidative stress measures in resting blood samples from baseline to 12 weeks. To assess the effects of yoga on oxidative stress, we measured intracellular total glutathione (GSH) levels and glutathione redox status, which is the ratio of reduced to oxidized glutathione (GSH:GSSG) as previously described [5] in red blood cells (RBCs) using the Thermo Scientific TSQ Quantum MAX, a triple quadrupole mass spectrometer (MS-MS) with electrospray mode (ESI). The liquid chromatography system consisted of Dionex Ultimate 3000 and a Zorbax Eclipse XDB C18 (3.0 × 150 mm × 3.0 μm) column. In addition, we measured malondialdehyde (MDA), a lipid peroxidation marker, using TBARS (TCA method) and protein carbonylation levels in plasma using commercial kits (Cayman Chemical, Ann Arbor, MI). Activity of intracellular antioxidant enzymes, superoxide dismutase (SOD), catalase, and glutathione peroxidase (GPx) was analyzed in RBCs using commercial colorimetric assay kits (Cayman Chemical, Ann Arbor, MI) as per manufacturer’s instructions.

Motor function was examined using the motor portion of the Unified Parkinson’s Disease Rating Scale (UPDRS), i.e., mUPDRS [24], which has high internal consistency and construct validity [25]. The mUPDRS includes a total of 14 items that provide 27 scores: speech, facial expression, body bradykinesia, posture, gait, and tremors. The scoring range for each item is from 0 (normal) to 4 (severe). Physical activity was measured by the 31-item Longitudinal Aging Study Amsterdam Physical Activity Questionnaire (LAPAQ) [26, 27]. The questionnaire is highly correlated with the 7-day diary (*r* = 0.68, *p* < .001) and moderately with the pedometer (*r* = 0.56, *p* < .001). The repeatability of the LAPAQ was reasonably good (weighted kappa, 0.65–0.75) in older adults [28].

Non-motor symptoms including cognitive function, mood, sleep quality, and quality of life were measured using standardized survey instruments. The MoCA [22] was used to assess participants’ cognitive function including domains of attention and concentration, executive function, conceptual thinking, calculations, visuospatial, memory, language, and orientation. This scale can detect mild cognitive impairment with 90–96% range sensitivity and specificity of 87% with 95% confidence interval [29].

The Beck Depression Inventory (BDI), a 21-item questionnaire, was used to measure participants’ characteristic attitudes and symptoms of depression [30]. A meta-analysis of the BDI’s internal consistency estimates yielded a mean coefficient alpha of 0.86 for psychiatric patients and 0.81 for non-psychiatric subjects [31].

Sleep quality was assessed by the Parkinson’s Disease Sleep Scale (PDSS) [32]. This 15-item questionnaire includes items that measure the overall quality of a night’s sleep, sleep onset and maintenance insomnia, nocturnal restlessness, nocturnal psychosis, nocturia, nocturnal motor symptoms, sleep refreshment, and daytime dozing. The scale demonstrated a high intraclass correlation coefficient (ICC) and a good discriminatory power between PD and healthy controls [32].

The 33-item Parkinson’s Disease Quality of Life Questionnaire (PDQUALIF) was used to assess quality of life in seven domains: social and role function, self-image/sexuality, sleep, outlook, physical function, independence, and urinary function, plus one item of global health-related quality of life [33]. Cronbach’s *α* of the scale was reported to be 0.89 and the ICC was 0.88 [33].

Feasibility was measured by the number of eligible subjects, number and type of yoga-related adverse events, retention rate, and reasons for not participating and for withdrawing from the study. Acceptability was evaluated at 12 weeks using an investigator-developed questionnaire. Participants were asked to rate their satisfaction through perceived enjoyment of class, the ease of class, and intention to continue use of the program using a 4-point Likert scale with “1” representing “not at all” and “4” representing “definitely.” Questions on whether participants were satisfied with the frequency and duration of the intervention program were also included. Program adherence was determined by class attendance during the intervention period and whether participants continued to practice yoga at 6-month post-intervention.

The follow-up survey was developed to examine the frequency and duration of yoga practice, factors that influence yoga adherence, beneficial yoga poses, and self-report PD symptoms 6 months after the intervention program. Participants received an email invitation that included a link to complete the follow-up survey via research electronic data capture (REDCap), a secure web application for building and managing online surveys and database [34].

Demographic information (e.g., age, race/ethnic background, education level, annual household income, marital status, and living arrangement), weight, height (for body mass index calculation), and comorbidities were collected from all participants.

### Data analysis

Descriptive statistics were presented as mean (SD) for continuous variables or count (percent) for categorical variables. All the clinical assessments were summarized by time points (at baseline, 12 weeks, and 6 months post-intervention). Data from the first baseline from the wait-list control group were used for between-group comparisons (baseline to 12 week). Data from the second baseline from the wait-list control were used for within-group analyses (12 to 24 weeks).

Data were assessed for whether they conformed reasonably to the normal distribution and to identify outliers. For unadjusted analysis of continuous variables, two-sample *t* tests were used for between-group comparisons and paired *t* tests for within-group comparisons; for categorical variables, Fisher’s exact test was used. To compare treatment effect between groups at 12 weeks, linear regression was applied adjusting for the corresponding baseline value and l-dopa; to assess the treatment effect within wait-list control group, a linear mixed model was applied adjusting for baseline and l-dopa value. Change scores (95% confidence interval for mean differences) were calculated for all the efficacy outcomes. All analyses used SAS (V9.4; SAS Institute, Cary, NC). No multiple comparison adjustment is done because this is a pilot study, and no definitive findings are claimed.

## Results

A total of 51 potential participants were initially screened by a trained RA over the telephone. Of these, 40 individuals met the inclusion criteria and underwent a second screening in person. Twenty individuals with PD who met all the study criteria and could commit to the dates and duration of the yoga intervention program were enrolled. The remaining individuals were excluded for a variety of reasons (Fig. 1).

Demographic characteristics and outcome data differences between the treatment and control groups at baseline are listed in Table 1. The participants had a mean age of 63 years ± 8 (49–75) and a mean disease duration of 4.8 years ± 2.9 (1–13). All participants had mild to moderate disease severity as determined by Hoehn and Yahr stages I–III [20] in their on medication state if they were taking PD medication. Eighteen (90%) participants were on dopaminergic medications.

**Table 1:** Baseline characteristics of the study participants

Variable	Yoga (*n* = 10)	Wait-list control (*n* = 10)
Demographics		
Age, years	63.5 (8.5)	65.8 (6.6)
White, *n* (%)	5 (50)	5 (50)
BMI, kg/m^2^	25.9 (2.6)	24.9 (3.3)
Education, *n* (%)		
12 years	0	1 (10)
13–16 years	7 (70)	4 (40)
≥ 17 years	3 (30)	5 (50)
Disease condition (range of scores)		
Hoehn and Yahr stage (I–III)	2 (.8)	2 (.8)
Duration of Parkinson’s disease, *n* (%)		
1–5 years	7 (70)	4 (40)
6–10 years	2 (20)	6 (60)
11–15 years	1 (10)	0
Comorbidities, *n* (%)		
0	0	2 (20)
1	9 (90)	5 (50)
≥ 2	1 (10)	3 (30)
Oxidative status		
Total GSH (μg/ml)	214.9 (38.3)	226 (39.4)
GSH:GSSG ratio	15.3 (8.1)	12.5 (1.0)
MDA (μM)	45.7 (11.8)	37.6 (9.6)
SOD (U/ml)	448.6 (161.7)	458.5 (129.1)
Catalase (nmol/min/ml)	36,709 (6186)	30,505 (3892)
Protein Carbonyl (nmol/ml)	20.5 (13.8)	28.4 (17)
GPx (nmol/min/ml/mg)	16.8 (4)	18.5 (5.4)
Motor function (range of scores)		
Motor UPDRS (0–108)	25.6 (6.9)	24.4 (7.3)
LAPAQ level, in minutes	5745 (4104)	7344 (3512)
Non-motor symptoms (range of scores)		
MoCA (0–30)	26.9 (2.2)	26.1 (2.4)
Beck Depression Inventory (0–63)	8.8 (5.9)	7.1 (5.0)
PD Sleep Scale (0–150)	112.3 (22.2)	107.2 (23.2)
PDQUALIF		
Social and role function (0–100)	37.5 (18.4)	35.3 (17.6)
Self-image and sexuality (0–100)	37.5 (17.1)	33.6 (18.8)
Sleep (0–100)	25.8 (20.2)	35.8 (24.6)
Outlook (0–100)	36.9 (17.5)	41.9 (12.9)
Physical functioning (0–100)	33.5 (12.3)	33.5 (16.3)
Independence (0–100)	10 (24.2)	16.3 (23.6)
Urinary function (0–100)	61.3 (22.4)	40 (17.5)
Global (0–100)	55 (15.8)	60 (17.5)

Values are the mean (SD) unless indicated otherwise

*UPDRS* Unified Parkinson’s Disease Rating Scale, *MoCA* Montreal Cognitive Assessment, *LAPAQ* Longitudinal Aging Study Amsterdam Physical Activity Questionnaire, *GSH* glutathione, *GSSG* glutathione disulfide, *MDA* malondialdehyde, *SOD* superoxide dismutase, *GPx* glutathione peroxidase, *PDQUALIF* Parkinson’s Disease Quality of Life Questionnaire

### Oxidative stress

Upon completion of the 12-week intervention program, we did not observe significant differences between the treatment and control groups in any of the seven variables (catalase, SOD, GPX, GSH, GSH:GSSH, MDA, and protein carbonylation) that measure oxidative stress status. Interestingly, we observed a decreasing trend in MDA, protein carbonylation, and SOD activity following intervention while total GSH and GPx showed an increasing trend, after adjusting for baseline and l-dopa values (Table 2). However, upon the completion of the program at 12 weeks, catalase level significantly increased following yoga intervention program compared to baseline (mean difference = 7779; 95% CI = 1280 to 14,278) in the immediate treatment group (Table 3).

**Table 2:** Between-group comparison of effects of yoga on oxidative status and motor and non-motor functions at 12 weeks adjusting for baseline measurement and l-dopa dose

Variable	Yoga	Wait-list control	Difference	95% CI
	(*n* = 10)	(*n* = 10)		
Oxidative status				
↑Total GSH (μg/ml)	211.4 (7.4)	207.5 (7.8)	− 3.9 (10.9)	(− 26.8, 19.0)
↑GSH:GSSG ratio	12.4 (.7)	13.1 (.7)	0.6 (1)	(− 1.5, 2.7)
↓MDA (μM)	42.7 (4.6)	46.5 (4.9)	3.8 (6.9)	(− 10.7, 18.3)
↓SOD (U/ml)	375.9 (37.5)	485.7 (39.5)	109.8 (55)	(− 6, 225)
↑Catalase (nmol/min/ml)	41,826 (3045)	42,583 (3249)	757 (4936)	(− 9613, 11,127)
↓Protein carbonyl (nmol/ml)	17 (2)	19.2 (2.1)	2.1 (3)	(− 4.2, 8.4)
↑GPx (nmol/min/ml/mg)	16.2 (1.6)	15.3 (1.7)	− 0.8 (2.3)	(− 5.6, 4.0)
Motor function				
↓Motor UPDRS (0–108)	17 (1.7)	22.5 (1.8)	5.4 (2.6)	(− 0.1, 10.9)
↑LAPAQ level, in minutes	2563 (756)	5749 (800)	3187 (1141)	(790, 5584)
QOS and QOL (range of scores)				
↑PD Sleep Scale (0–150)	112.2 (4.1)	106.3 (4.3)	− 5.8 (6)	(− 18.4, 6.8)
↑MoCA (0–30)	28.1 (.4)	27.5(.4)	− 0.8 (.6)	(− 2.1, 0.5)
↓Beck Depression Inventory (0–63)	8.9 (1.1)	8.6 (1.2)	− 0.3 (1.7)	(− 3.9, 3.3)
↓PDQUALIF				
Social and role function (0–100)	44 (3.8)	41.8 (4)	−2.2 (5.6)	(−14.0, 9.6)
Self-image and sexuality (0–100)	37.6 (3.9)	41.5 (4.1)	3.8 (5.8)	(− 8.4, 16.0)
Sleep (0–100)	35.1 (3.1)	24.7 (3.3)	− 10.4 (4.6)	(− 20.1, − 0.7)
Outlook (0–100)	45.8 (2.9)	35.2 (3)	− 10.6 (4.3)	(− 19.6, − 1.6)
Physical functioning (0–100)	33.9 (3.2)	36.2 (3.4)	2.3 (4.7)	(− 7.6, 12.2)
Independence (0–100)	10 (5)	5.4 (5.3)	− 4.5 (7.4)	(− 20.0, 11.0)
Urinary function (0–100)	50.8 (4.9)	47.7 (5.3)	− 3 (7.7)	(− 19.2, 13.2)
Global (0–100)	52 (6.3)	53.3 (6.6)	1.2 (9.3)	(− 18.3, 20.7)

Values are the mean (SE) unless indicated otherwise. ↑or ↓sign indicates better status

*CI* confidence interval, *UPDRS* Unified Parkinson’s Disease Rating Scale, *MoCA* Montreal Cognitive Assessment, *LAPAQ* Longitudinal Aging Study Amsterdam Physical Activity Questionnaire, *GSH* glutathione, *GSSG* glutathione disulfide, *MDA* malondialdehyde, *SOD* superoxide dismutase, *GPx* glutathione peroxidase, *PDQUALIF* Parkinson’s Disease Quality of Life Questionnaire

**Table 3:** Within-group comparison of yoga effects

Variable	Baseline	12 weeks	Difference	95% CI
	(*n* = 10)	(*n* = 10)		
Oxidative status				
↑Total GSH (μg/ml)	214.1 (38.3)	207.5 (35.4)	− 7.4 (38.4)	(− 34.9, 20.1)
↑GSH:GSSG ratio	15.3 (8.1)	12.5 (1.8)	− 2.7 (8.73)	(− 8.9, 3.5)
↓MDA (μM)	45.7 (11.8)	45.7 (18.9)	0.06 (16.3)	(− 11.6, 11.7)
↓SOD (U/ml)	448.6 (161.7)	384.1 (99.8)	− 64.5 (220.1)	(− 222, 93)
↑Catalase (nmol/min/ml)	36,709 (6186)	44,488 (9212)	7779 (9085)	(1280, 14,278)
↓Protein carbonyl (nmol/ml)	20.5 (13.8)	15.3 (6.4)	− 5.1 (12.4)	(− 14.0, 3.8)
↑GPx (nmol/min/ml/mg)	16.8 (4)	16.4 (4.6)	− 0.4 (5)	(− 4.0, 3.2)
Motor function				
↓Motor UPDRS (0–108)	25.6 (6.9)	17.5 (5.3)	− 8.1 (6.4)	(− 12.7, − 3.5)
↑LAPAQ level, in minutes	5745 (4104)	2608 (1286)	− 3137.8 (4353)	(− 6252, − 24)
QOS and QOL (range of scores)				
↑PD Sleep Scale (0–150)	112.3 (22.2)	112.9 (17.4)	0.6 (11.9)	(− 7.9, 9.1)
↑MoCA (0–30)	26.9 (2.2)	28.4 (1.3)	1.5 (2)	(0.1, 2.9)
↓Beck Depression Inventory (0–63)	8.8 (5.9)	9.7 (5.7)	0.9 (3.6)	(− 1.7, 3.5)
↓PDQUALIF				
Social and role function (0–100)	37.5 (18.4)	46.4 (21.1)	8.9 (10.7)	(1.2, 16.6)
Self-image and sexuality (0–100)	37.5 (17.1)	42.5 (19.2)	5 (13.8)	(− 4.9, 14.9)
Sleep (0–100)	25.8 (20.2)	31.7 (17)	5.8 (6.9)	(0.9, 10.7)
Outlook (0–100)	36.9 (17.5)	43.8 (17.4)	6.9 (8.6)	(0.7, 13.1)
Physical functioning (0–100)	33.5 (12.3)	34.5 (10.4)	1 (12.4)	(− 7.9, 9.9)
Independence (0–100)	10 (24.2)	10 (16.5)	0 (25.7)	(− 18.4, 18.4)
Urinary function (0–100)	61.3 (22.4)	56.3 (18.9)	− 5 (14.7)	(− 15.5, 5.5)
Global (0–100)	55 (15.8)	50 (23.6)	− 5 (19.7)	(− 19.1, 9.1)

Values are the mean (SD) unless indicated otherwise. ↑or ↓sign indicates better status

*CI* confidence Interval, *UPDRS* Unified Parkinson’s Disease Rating Scale, *MoCA* Montreal Cognitive Assessment, *LAPAQ* Longitudinal Aging Study Amsterdam Physical Activity Questionnaire, *GSH* glutathione, *GSSG* glutathione disulfide, *MDA* malondialdehyde, *SOD* superoxide dismutase, *GPx* glutathione peroxidase, *PDQUALIF* Parkinson’s Disease Quality of Life Questionnaire

### Motor function

Compared to the participants in the control group at 12 weeks, participants’ motor functions in the treatment group based on the mUPDRS scores were significantly better (mean difference = 5.4; 95% CI = − 0.1 to 10.9) (Table 2). These participants also demonstrated a significant improvement in motor functions compared to their baseline score (mean difference = − 8.1; 95% CI = − 12.7 to − 3.5) (Table 3).

The treatment group’s LAPAQ score at 12 weeks was found to be significantly lower compared to that of the control group (mean difference = 3187; 95% CI = 790 to 5584) (Table 2). Based on the within-group comparison, the score was also found to be significantly lower (mean difference = − 3137.8; 95% CI = − 6252 to − 24) at 12 weeks compared to that before the program began (Table 3).

### Non-motor functions

At 12 weeks, sleep quality, depression level, cognitive function, and global quality of life were not significantly different between the two groups. Compared to participants in the control group, two domains of quality of life as measured by the PDQUALIF Scale were significantly worse among the participants in the treatment group: sleep (mean difference = − 10.4; 95% CI = − 20.1 to − 0.7) and outlook (mean difference = − 10.6; 95% CI = − 19.6 to − 1.6) (Table 2). In within-group comparison, sleep (mean difference = 5.8; 95% CI = 0.9 to 10.7) and outlook (mean difference = 6.9; 95% CI = 0.7 to 13.1) along with another domain, social and role function (mean difference = 8.9; 95% CI = 1.2 to 16.6), were also worse than before the program began (Table 3). However, compared to those before the program began, participants in the treatment group scored significantly higher in the cognitive function (mean difference = 1.5; 95% CI = 0.1 to 2.9) at 12 weeks based on their MoCA scores (Table 3).

### Feasibility/acceptability

It took 4 months to recruit the desired number of participants. The program retention rate was 95%. One participant had to drop out before the study began due to pneumonia. No yoga-related adverse events were reported during the study.

Among the 19 participants who completed the intervention program, 17 (89%) rated that they were “definitely” satisfied with the program at the end of the intervention period. Many participants were satisfied with the frequency (*n* = 12, 63%) and duration (*n* = 14, 74%) of the program. Participants who were somewhat dissatisfied recommended that the program to be offered 3 days a week for 24–39 weeks.

### Program adherence

Seventeen (85%) of the participants attended at least 75% of the classes and four participants attended 100%. The average attendance rate was 80% per class. Only two participants missed more than 75% of class, both due to issues that were unrelated to the yoga intervention (*Clostridium difficile* infection and transportation problems).

At 6 months post-intervention, a follow-up survey via REDCap was sent to the 19 participants who completed the study. The response rate was 74% (*n* = 14). Among the participants who completed the online survey, six participants (43%) signed up for a yoga class and four (29%) continued to practice yoga independently at home. The average number of days per week that the respondents reported to be practicing yoga was 2 ± 1.5 (1–4) days. The average number of minutes each time they practiced yoga was 32.5 ± 31 (5–75) min. The reasons for continuing practicing yoga reported by most respondents (75%) were for managing motor symptoms and improving strength and balance. Seven (50%) respondents indicated that they incorporated yoga “all of the time” or “some of the time” in their exercise routine. Health problems were the main reasons for respondents to neither practice yoga independently (40%) nor sign up for a yoga class (50%). Those who reported not practicing yoga, 80% would consider practicing again in the future.

## Discussion

The results demonstrate that yoga is safe, feasible, and acceptable in individuals with mild-moderate PD. Yoga may serve as a complementary method for improving motor function. However, higher frequency and longer duration may be necessary for it to positively impact oxidative stress level and non-motor functions in this population.

Contrary to previous research that found regular yoga practices were able to improve antioxidant and oxidative status and reduce oxidative stress in young and healthy adults or individuals with other type of chronic health conditions [12–15], findings from our between-group analysis suggest that twice weekly 60-min group-based yoga for 12 weeks was not able to produce a significant change in oxidative status in individuals with PD. We did not observe significant differences in most of the antioxidant enzymes. Factors such as the initial status prior to training (older adults with a neurodegenerative condition), training protocol (frequency and duration), and the time of administering PD medication prior to blood collection could have affected the results [35]. Additionally, because physical activity has been related to lipid peroxidation and antioxidant enzyme levels [36], the higher physical activity level among the participants in the wait-list control group compared to the ones in the immediate treatment group (based on the LAPAQ score) might have contributed to the outcome. However, there was a decreasing trend in lipid peroxidation as indicated by MDA and protein carbonylation, which are indicative of reduced oxidative stress following yoga.

The increased catalase activity following Hatha Yoga within the immediate treatment group could be beneficial for PD, especially because MDA, protein carbonyl, and GSH:GSSG were not significantly altered. This indicates that our yoga regimen can specifically improve the antioxidant enzyme capacity without altering the overall oxidative status in these individuals. However, we did not observe a similar increase in between-group comparison, which may be due to the significant difference in catalase activity prior to the intervention program. This needs to be further investigated. Future studies with a larger sample size, higher frequency of yoga practice, longer program duration, and the specific time when participants take their medications relative to blood collection will be needed to determine the specific impact of yoga on the biochemical markers that indicate the body’s stress response.

Our findings showing improved motor function are consistent with the current literature on the effect of yoga on PD [16–19]. Because the current conventional pharmacologic interventions for PD are limited, medications can cause unwanted side effects and become less effective over time [37]. Yoga may be a safe and effective complementary method for managing motor symptoms, improving motor function, and preventing functional deterioration in PD.

Overall, participants were not more physically active after practicing yoga. This finding is consistent with previous reports in adults with or at risk for type II diabetes [38] and women with symptoms of posttraumatic stress disorder [39] but contradicts a report in women with multiple sclerosis [40]. It is uncertain whether participants in the treatment group had a lower energy level or they substituted their regular physical activity with yoga. Also, it is unknown if those in the wait-list control group increased their physical activity because of their study participation prior to undergoing the yoga intervention.

Although a small number of studies found yoga to be beneficial to psychological well-being and aspects of quality of life in PD [16, 18], current evidence on the non-motor benefits of yoga in PD remains unclear. The finding on the effect of yoga on sleep is inconclusive. Although the sleep quality domain in PDQUALIF was reported to be significantly worse in the immediate treatment group compared to the wait-list control group at 12 weeks, there were no significant differences in the PD sleep scores between groups. Factors that affect sleep quality and timing/dose of yoga needed for promoting sleep quality warrant further investigation. “Outlook” was also reported to be significantly worse among participants in the treatment group. A possible reason could be that when people begin the journey of becoming more mindful and aware of their lives, the areas where there is unhappiness that they might have been ignoring sometimes come to the forefront of awareness [41]. Because practicing positive thoughts and self-acceptance are part of yoga teaching, a longer intervention program may affect participants’ outlook differently.

Participants’ cognitive function was found to be significantly improved within group but not when compared with the control group. The improved cognition within group might be due to a test-retest effect of the MoCA test. Outcomes of non-motor symptoms such as sleep quality, depression level, and quality of life were not different between the two groups. However, the effect sizes for the differences in these measures will be used to plan future studies.

The favorable class attendance, the high program satisfaction ratings, and the desirable yoga adherence rate after the intervention suggest the enthusiasm that PD participants may have for yoga. Considering the popularity of yoga among the general population and the wide availability and utilization of yoga practice in the community, evidence-based data from larger trials are needed for health care providers to communicate and educate their PD patients about the use of yoga for PD.

### Limitations

There are several study limitations. The small sample size of this study may have affected the study findings and limits their generalizability. However, results from this pilot study will lead to a better-designed RCT and serve as a discussion point about setting a threshold value for calculating the sample size. Due to safety reasons, the exclusion of independent home yoga practice might have limited the therapeutic effects. Results of this pilot program demonstrated that the yoga intervention program is safe and acceptable, and future studies should consider including adding a home-based yoga regime designed for the unique needs of the PD population. This may be beneficial for enhancing yoga’s therapeutic effects. In spite of these limitations, our study has several notable strengths including the strong content validity of the program designed specifically for PD established with an expert panel of yoga teachers, use of a RCT design, and the inclusion of the longer post-intervention follow-up period.

## Conclusions

Findings suggest that yoga is feasible and acceptable. It may serve as a complementary method for improving motor function in PD. Further research in larger samples is needed to determine its impact on oxidative stress and non-motor symptoms.

## Abbreviations

BDI: Beck Depression Inventory
GPx: Glutathione peroxidase
GSH: Glutathione
GSSG: Glutathione disulfide
ICC: Intraclass correlation coefficient
ID: Identification
LAPAQ: Longitudinal Aging Study Amsterdam Physical Activity Questionnaire
MDA: Malondialdehyde
MoCA: Montreal Cognitive Assessment
mUPDRS: Motor portion of Unified Parkinson’s Disease Rating Scale
PD: Parkinson’s disease
PDQUALIF: Parkinson’s Disease Quality of Life Questionnaire
PDSS: Parkinson’s Disease Sleep Scale
RBCs: Red blood cells
RCT: Randomized controlled trial
REDCap: Research electronic data capture
SD: Standard deviation
SOD: Superoxide dismutase
